# First-year outcomes of very low birth weight preterm singleton infants with hypoxemic respiratory failure treated with milrinone and inhaled nitric oxide (iNO) compared to iNO alone: A nationwide retrospective study

**DOI:** 10.1371/journal.pone.0297137

**Published:** 2024-05-09

**Authors:** Ya-Ting Chang, Jia-Rou Liu, Wei-Min Chen, Chi-Nan Tseng, Lai-Chu See

**Affiliations:** 1 Department of Pediatrics, Chang Gung Memorial Hospital at Linkou, Taoyuan City, Taiwan; 2 Department of Public Health, College of Medicine, Chang Gung University, Taoyuan City, Taiwan; 3 Department of Cardiac Surgery, Chang Gung Memorial Hospital at Linkou, Taoyuan City, Taiwan; 4 Division of Rheumatology, Allergy and Immunology, Chang Gung Memorial Hospital at Linkou, Taoyuan City, Taiwan; 5 Biostatistics Core Laboratory, Molecular Medicine Research Center, Chang Gung University, Taoyuan City, Taiwan; Saitama Medical Center: Saitama Ika Daigaku Sogo Iryo Center, JAPAN

## Abstract

**Background:**

Inhaled nitric oxide (iNO) has a beneficial effect on hypoxemic respiratory failure. The increased use of concurrent iNO and milrinone was observed. We aimed to report the trends of iNO use in the past 15 years in Taiwan and compare the first-year outcomes of combining iNO and milrinone to the iNO alone in very low birth weight preterm (VLBWP) infants under mechanical ventilation.

**Methods:**

This nationwide cohort study enrolled preterm singleton infants with birth weight <1500g treated with iNO from 2004 to 2019. Infants were divided into two groups, with a combination of intravenous milrinone (Group 2, n = 166) and without milrinone (Group 1, n = 591). After propensity score matching (PSM), each group’s sample size is 124. The primary outcomes were all-cause mortality and the respiratory condition, including ventilator use and duration. The secondary outcomes were preterm morbidities within one year after birth.

**Results:**

After PSM, more infants in Group 2 needed inotropes. The mortality rate was significantly higher in Group 2 than in Group 1 from one month after birth till 1 year of age (55.1% vs. 13.5%) with the adjusted hazard ratio of 4.25 (95%CI = 2.42–7.47, p <0.001). For infants who died before 36 weeks of postmenstrual age (PMA), Group 2 had longer hospital stays compared to Group 1. For infants who survived after 36 weeks PMA, the incidence of moderate and severe bronchopulmonary dysplasia (BPD) was significantly higher in Group 2 than in Group 1. For infants who survived until one year of age, the incidence of pneumonia was significantly higher in Group 2 (28.30%) compared to Group 1 (12.62%) (p = 0.0153).

**Conclusion:**

Combined treatment of iNO and milrinone is increasingly applied in VLBWP infants in Taiwan. This retrospective study did not support the benefits of combining iNO and milrinone on one-year survival and BPD prevention. A future prospective study is warranted.

## Introduction

Over the past few decades, advancements in the medical care of preterm infants have improved the survival outcomes of very low birth weight infants and infants born as early as the gestational age of 23 weeks. These highly premature infants are vulnerable to disrupting alveolarization and insulting pulmonary vascular growth. Providing adequate mechanical ventilation for respiratory failure with minimal lung injury in this vulnerable group remains challenging for clinicians. Despite most very preterm and extremely preterm neonates requiring mechanical ventilation, the consensus on optimal management of hypoxemic respiratory failure among these preterm neonates is still lacking.

Nitric oxide (NO) is an endothelial-derived pulmonary vasodilator. NO mediates intracellular increase of cyclic guanosine monophosphate (cGMP) and facilitates vascular smooth muscle cell relaxation [1]. Since its introduction in routine clinical practice in 1988 [2], inhaled NO (iNO) has been established to treat refractory hypoxemic respiratory failure and persistent pulmonary hypertension of newborns in term and late preterm infants [3]. For preterm infants with a birth weight of less than 1500 g, iNO increases the partial pressure of arterial oxygen, whereas the incidence of death and bronchopulmonary dysplasia was not reduced. Early use of iNO has been proposed to prevent bronchopulmonary dysplasia, but the randomized clinical trial fails to show the benefit [4]. Overall, iNO is still controversial for hypoxemic respiratory failure in preterm infants. In Taiwan, there is no consensus on iNO therapy in preterm infants, but the expense of iNO therapy is covered by National Health Insurance. The clinicians may start using iNO for infants with hypoxemic respiratory failure under maximal ventilator support and have an oxygen index> 25 in two consecutive arterial blood gases taken 30 minutes apart.

Multiple factors contribute to hypoxemic respiratory failure in preterm infants, including parenchymal lung diseases (such as pulmonary hypoplasia, respiratory distress syndrome, pneumonia), cardiac disorders (especially in right ventricular systolic dysfunction as well as left ventricular diastolic dysfunction), and pulmonary hypertension resulting from increased pulmonary vascular resistance [5]. However, making a differential diagnosis in clinical practice is difficult because the factors above may exist concurrently. Therefore, the use of inotropes is often considered in this vulnerable group.

Milrinone is a synthetic non-catecholamine indicator that inhibits phosphodiesterase type III, resulting in increased cyclic adenosine monophosphate (cAMP) levels and pulmonary vasculature vasodilation [6]. Milrinone has been used to treat preterm infants with hemodynamic instability [7] and post-ligation syndrome following the obliteration of the ductus arteriosus [8]. Recent studies have also reported that milrinone plays a role in treating pulmonary hypertension in neonates [9, 10], especially for those with suboptimal response to iNO [11].

In 2014, the American Academy of Pediatrics reported that the existing evidence does not support routine iNO use in preterm infants. However, the off-label use of iNO on maximal ventilatory support has risen because there is no effective treatment for refractory hypoxemic respiratory failure in preterm infants [12, 13]. A specific subgroup of very low birth weight preterm (VLBWP) infants with pulmonary hypertension may benefit from iNO. In clinical practice, echocardiography could be a helpful screening tool for infants with pulmonary hypertension. However, right heart catheterization is still a golden diagnosis standard and is challenging to perform in VLBWP infants [14]. It has been shown that a combination of iNO and milrinone infusion has an additive effect on lowering pulmonary vascular resistance [15]. The combined use of iNO and milrinone for refractory hypoxemic respiratory failure in neonatal intensive care units has gained some use. However, the benefits of long-term outcomes have not been documented.

This nationwide retrospective cohort study aimed to report the trends of iNO use with/without milrinone in the past 15 years in Taiwan and to examine whether cotreatment of iNO and milrinone can improve the first-year outcomes in VLBWP infants undergoing mechanical ventilation compared to iNO treatment. The primary outcomes were all-cause mortality and the respiratory condition, including ventilator support use and duration. The secondary outcomes were preterm-associated morbidities within one year after birth.

## Materials and methods

### Data source

Data were obtained from four nationwide databases: the Taiwan Birth Registry (TBR), Taiwan Maternal and Child Health Database (TMCHD), National Health Insurance Research Database (NHIRD), and Taiwan Death Registry (TDR). All records are available from the Health and Welfare Data Center (HWDC). All personal identification numbers in the TMCHD, NHIRD, and TDR were scrambled using the same method so that they were allowed to link together. Only summarized results are allowed to be released from the HWDC to protect privacy further.

**Fig 1:**
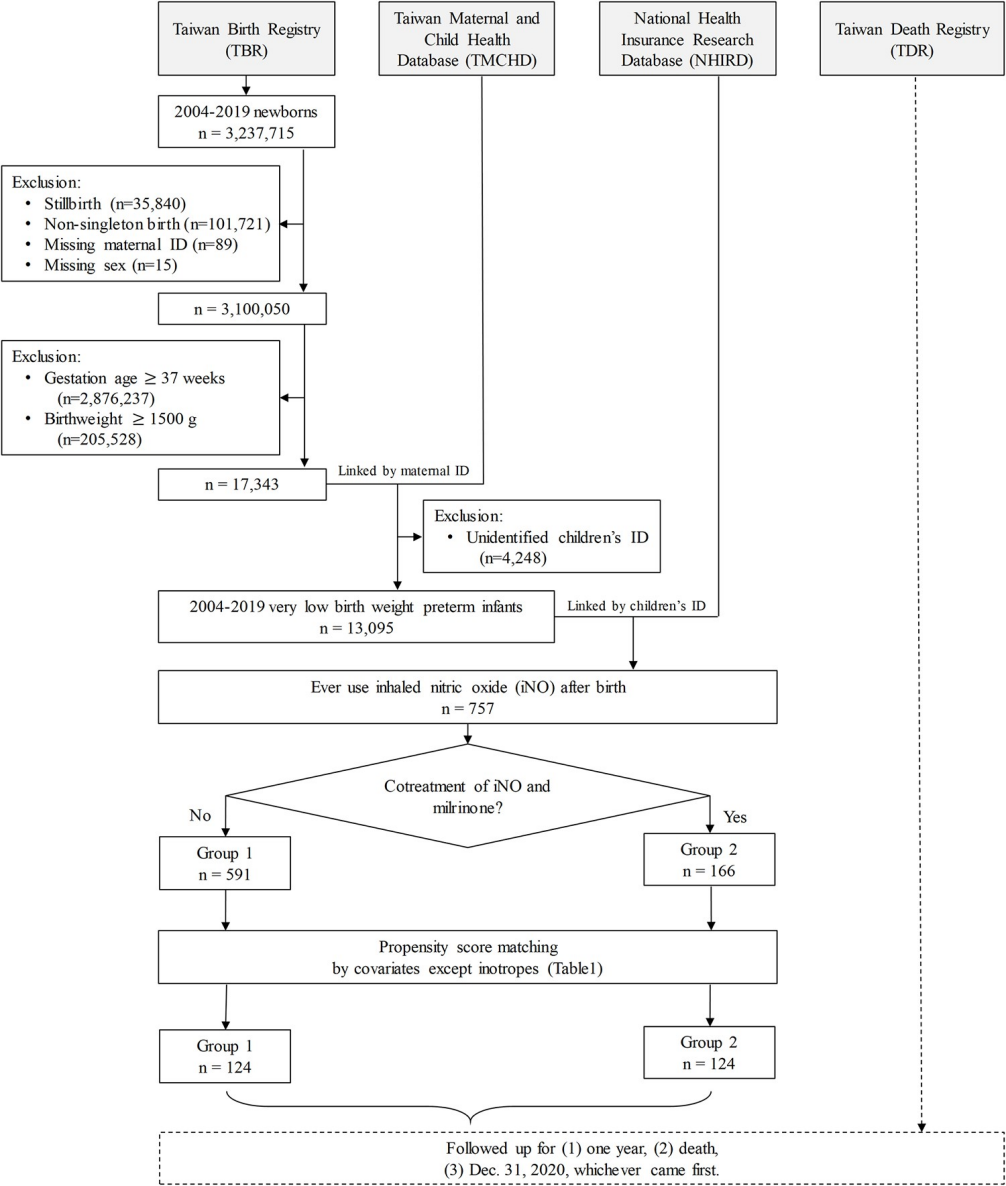
The flowchart to enroll very low birth weight preterm infants treated with inhaled nitric oxide.

As shown in **Fig 1**, we linked the TBR and TMCHD by the same maternal ID. The TMCHD contained parents’ and children’s IDs, allowing the link between the medical claims of offspring and their parents. Almost all (99.78%) TBR births correspond to NHIRD and TMCHD records [16]. The TBR contains sex, gestational age, birth weight, Apgar score at 1 min and 5 min, whether the birth was live or stillbirth, and maternal age. Previous studies have reported a low rate of missing information and high validity of the TBR compared with obstetric records [17].

Next, we linked the TBR, TMCHD, NHIRD, and TDR by the children’s ID (**Fig 1**). The NHIRD is a claim database from the Taiwan National Health Insurance Program founded in 1995 and covers 99.9% of the Taiwanese population. The NHIRD contains beneficiaries, ambulatory care claims, inpatient claims, prescriptions dispensed at pharmacies, and the registry for board-certified specialists. The NHIRD uses the International Classification of Diseases, Ninth Edition, Clinical Modification (ICD-9-CM) before 2016, and the Tenth Edition (ICD-10-CM) after 2016. The validation of different diagnostic codes in the NHIRD has been reported [18].

The TDR details the date of death and the underlying cause (ICD-9-CM code and ICD-10-CM code since 2008). Taiwan’s data on death certificates are considered highly accurate and complete [19].

#### Ethics statement

The study was approved by the Institutional Review Board of the Chang Gung Medical Foundation (201901071B0). Informed consent was waived because all personal identification information was encrypted.

### Study design and study groups

This is a nationwide retrospective cohort study. The inclusion criteria were singleton birth, gestational age <37 weeks, birthweight <1500g, and alive after birth. Records without maternal ID or sex were excluded. VLBWP infants receiving iNO were divided into two groups, with a combination of intravenous milrinone (Group 2) and without milrinone (Group 1). Group 2 required that intravenous milrinone should be initiated on the same day or no later than the first three days of iNO therapy, and the difference in duration between milrinone and iNO therapy should be no more than seven days.

### Outcomes, covariates, and follow-up

The primary outcomes were all-cause mortality and the duration of ventilator support during the first year after birth. Invasive mechanical ventilation was defined as endotracheal tube intubation with conventional mechanical ventilation. In addition, data on other types of mechanical ventilation support (e.g., high-frequency oscillatory ventilation (HFOV), non-invasive positive pressure ventilation, supplemental oxygen therapy with nasal cannulas), and bronchopulmonary dysplasia (BPD) were also retrieved. The definition of BPD and the classification of moderate and severe BPD were determined based on the NICHD revised criteria [20]. Secondary outcomes were prematurity-related morbidities, including intraventricular hemorrhage (IVH), necrotizing enterocolitis (NEC), epilepsy, late-onset sepsis, pneumonia, and retinopathy of prematurity (ROP). Other than the ICD-9-CM code or ICD-10-CM code, hospitalization once or outpatient visits at least twice is required to reduce misclassification (**S1 Table**).

Other covariates included sex, gestational age, birth weight, Apgar scores 1 and 5 minutes after birth, patent ductus arteriosus (PDA) and PDA ligation, atrial septal defect (ASD), perinatal bacterial sepsis, Survanta use, dobutamine use, dopamine use, and epinephrine use. The VLBWP infants were categorized into small for gestational age (SGA, birth weight below 10^th^ percentile of the gestational week), appropriate for gestational age (AGA), and large for gestational age (LGA, birth weight greater than 90^th^ percentile of the gestational week) [21]. All VLBWP infants receiving iNO were followed up for one year until December 2020, or the date of death, whichever came first. The ICD-9-CM and ICD-10-CM codes for diagnosis, procedures, and medication code used in this study are listed in **S1 and S2 Tables.**

### Statistical analysis

To reduce the confounding effect in observational studies, propensity score matching (PSM) was performed to balance the birth characteristics between the two study groups [22]. The two study groups were matched in a 1:1 ratio within a caliper width equal to 0.2, the standard deviation (SD) of the logit of the estimated propensity score. The birth characteristics (**Table 1,** except inotropes use) were included in the generalized boosted model (GBM) to obtain the propensity score. The GBM is an iterative process with multiple regression trees to capture complex and nonlinear relationships of covariates to achieve an optimal balance between the two study groups [23]. The advantages of GBM are less affected by extreme weights and model misspecification [24].

**Table 1:** The birth characteristics, clinical data, and inotropic use among very low birth weight preterm infants treated with nitric oxide before and after propensity score matching (PSM).

	Before propensity score matching			After propensity score matching		
	Group 1 (n = 591)	Group 2 (n = 166)	P	Group 1 (n = 124)	Group 2 (n = 124)	P
Male	334 (56.51%)	93 (56.02%)	0.910	65 (52.42%)	69 (55.65%)	0.610
Gestational age (week)			0.508			0.014
Mean ± SD	26.93±2.58	26.78±2.45		25.93±1.96	26.64±2.50	
Median ± IQR	27±4	26±3		26±3	26±3	
Birth weight (g)			<0.001			0.002
Mean ± SD	922.37±263.35	809.57±264.10		779.16±232.02	871.77±243.92	
Median ± IQR	870±396	764±340		725±276	833±348	
Small for gestational age	36 (6.09%)	36 (21.69%)	<0.001	11 (8.87%)	5 (4.03%)	0.121
Apgar score at 1 min			0.300			0.948
Median ± IQR	5±3	5±3		5±2	5±3	
Apgar score at 5 min			0.614			0.737
Median ± IQR	7±2	7±2		8±2	7±2	
Patent ductus arteriosus (PDA)	253 (42.81%)	96 (57.83%)	0.001	82 (66.13%)	67 (54.03%)	0.052
Prophylactic PDA ligation	137 (54.15%)	53 (55.21%)	0.859	48 (58.54%)	40 (59.70%)	0.288
Atrial septal defect (ASD)	35 (5.92%)	18 (10.84%)	0.028	7 (5.65%)	10 (8.06%)	0.451
Perinatal bacterial sepsis	179 (30.29%)	42 (25.30%)	0.212	33 (26.61%)	35 (28.23%)	0.776
Prophylactic Survanta use	209 (35.36%)	44 (26.51%)	0.033	34 (27.42%)	32 (25.81%)	0.774
Inotropes use						
Dobutamine use	174 (29.44%)	99 (59.64%)	<0.001	35 (28.23%)	70 (56.45%)	<0.001
Dopamine use	307 (51.95%)	139 (83.73%)	<0.001	79 (63.71%)	104 (83.87%)	0.001
Epinephrine use	356 (60.24%)	144 (86.75%)	<0.001	83 (66.94%)	105 (84.68%)	0.001

Group 1, without milrinone use; Group 2, with milrinone use; IQR, interquartile range; SD, standard deviation.

Categorical data (such as the Apgar scores, SGA, and PDA) and continuous data (such as the gestational weeks and birth weight) were compared using the Chi-square test, Fisher’s exact test, or independent t-test, where appropriate. For IVH and NEC study outcomes, the incident rate was defined as the number of events divided by the number of VLBWP infants. Logistic regression was performed to obtain the odds ratios (ORs) with 95% confidence intervals (CIs) of IVH and NEC for Group 2 when compared with Group 1 (**Table 3**). Besides the study grouping, inotropic use (dobutamine, dopamine, and epinephrine) was included in the logistic model because the two study groups observed a significant difference in inotropic use.

For study outcomes that occurred during the first year (such as epilepsy, late-onset sepsis, pneumonia, and ROP), the incident rate of the outcome was the number of events divided by the number of VLBWP infants. Cox’s proportional hazard model was used to obtain the hazard ratios (HRs) with 95% CI for Group 2 compared with Group 1. Again, the use of inotropes was included in Cox’s model because there was a significant difference in inotropic use between the two study groups. Because of the high mortality among the VLBWP infants, the subdistribution hazard ratio (SHR) and its confidential interval (CI) for the study outcomes were obtained using the Fine-Gray model, which accounts for the competing risk of death [25]. A *P* value <0.05 was considered significant.

## Results

First, we identified 3,237,715 newborns from 2004–2019, of which 13,095 (around 4‰) were preterm singleton births (gestational age < 37 weeks) with very low birth weight (< 1,500 g) from TBR. Second, these records were matched with NHIRD and TMCHD to determine whether these newborns received iNO after birth (n = 757, 5.78%). Finally, the 757 newborns treated with iNO were divided into two groups depending on the use of milrinone at the time of iNO use: Group 1 (iNO without milrinone, n = 591) and Group 2 (iNO with milrinone, n = 166) **(Fig 1)**.

### The trends of iNO use in VLBWP infants from 2004 to 2019

**Fig 2:**
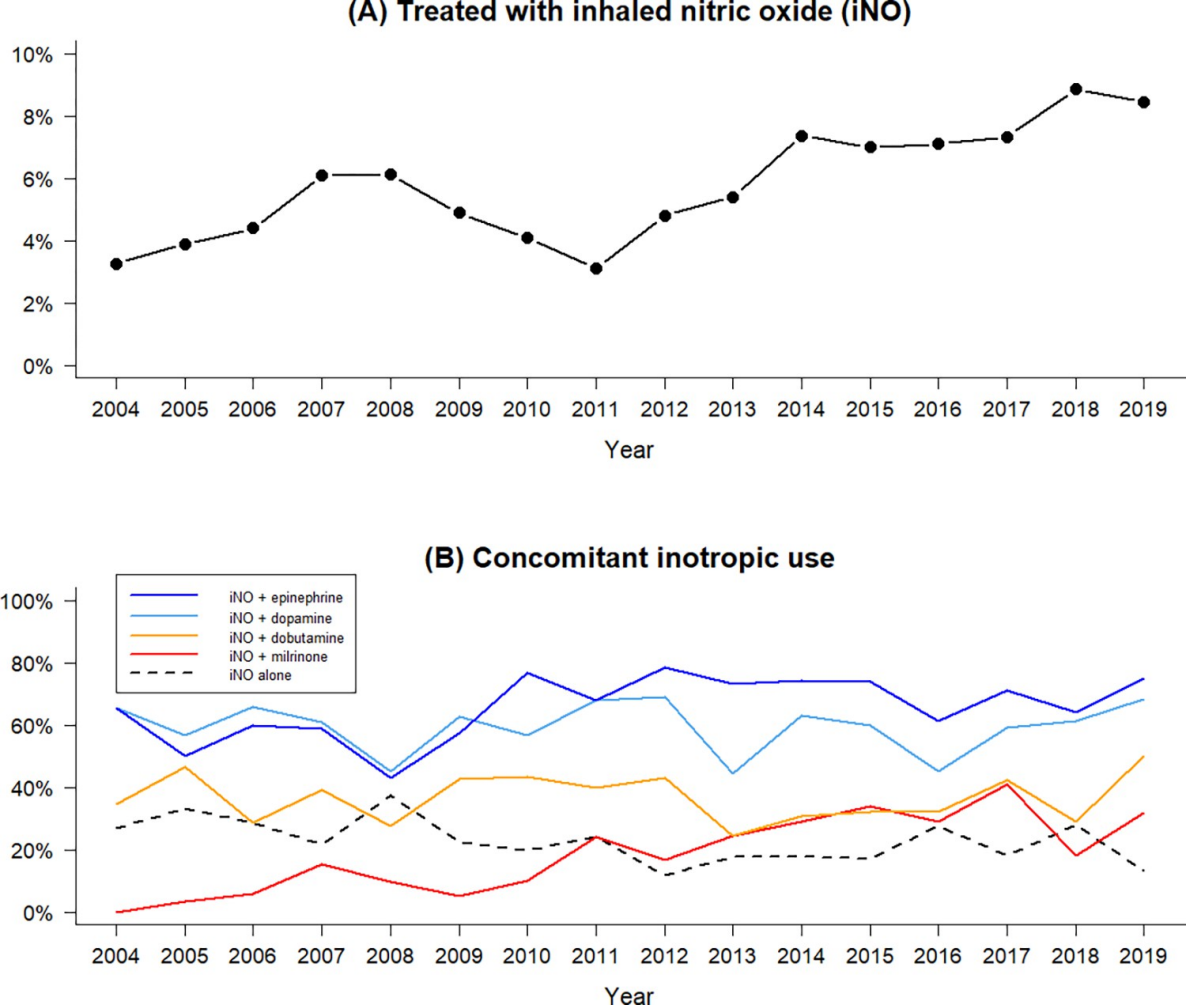
Treated with inhaled nitric oxide (iNO) (A) and concomitant inotropic use (B) among very low birth weight preterm infants in Taiwan, 2004–2019.

Among the 757 VLBWP infants who received iNO, 511 (67.5%) had birth weights below 1000 g, and 488 (64.4%) were born at gestational age less than 28 weeks. In 2004, 3.3% of VLBWP infants were treated with iNO for hypoxemic respiratory failure, gradually increasing to 8.4% in 2019. Inotropes were frequently used during iNO treatment (**Fig 2A**). Only one in four VLBWP infants used iNO without inotropic support. Epinephrine and dopamine were the most used inotropes with iNO. Of note, the concurrent treatment of iNO and milrinone steadily increased from 0.0% to 31.7% during 2004–2019 **(Fig 2B**).

### Patient characteristics

**Fig 3:**
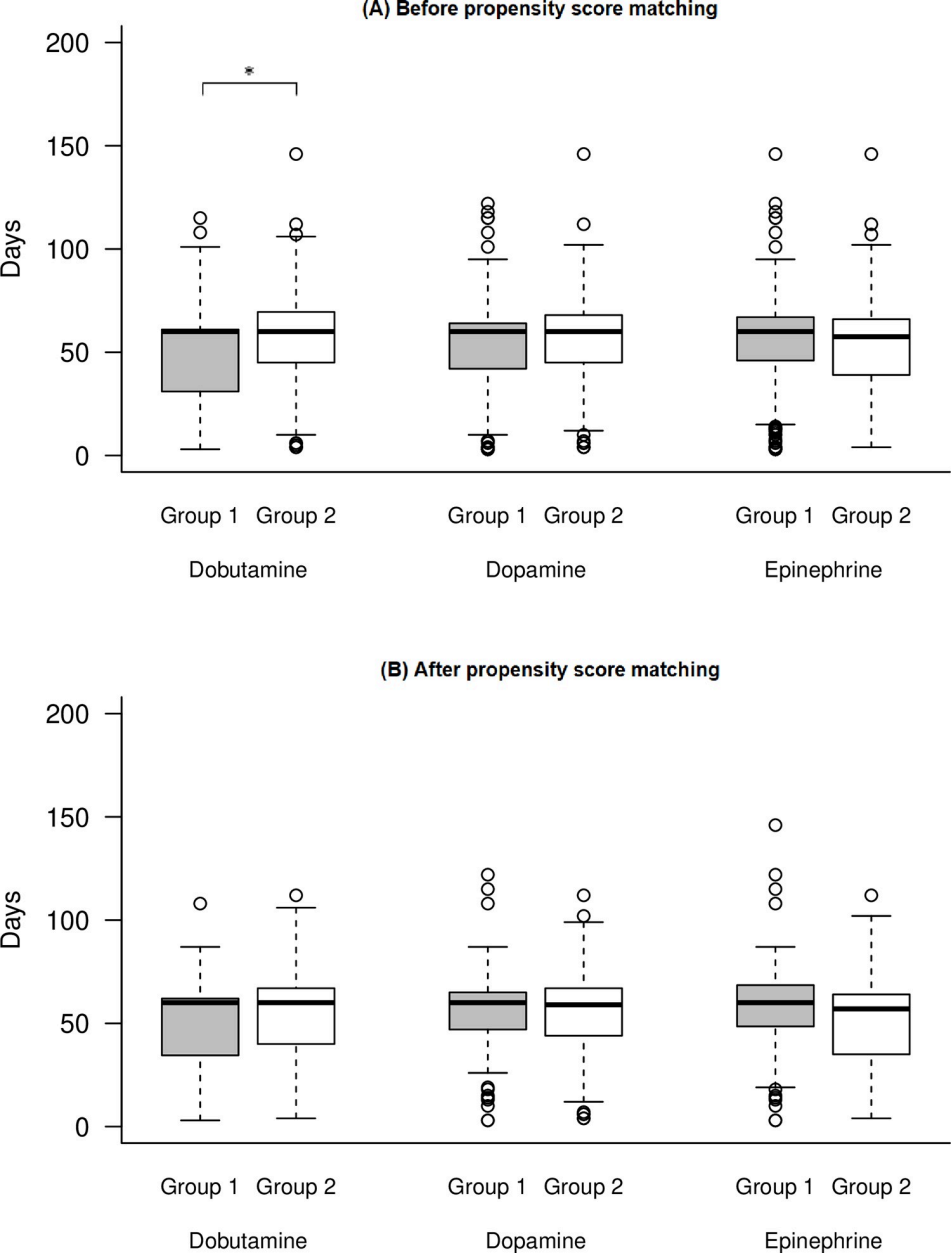
The duration of inotropic use (days) between Group 1 and Group 2 of very low birth weight preterm (VLBWP) infants treated with nitric oxide, (A) before and (B) after propensity score matching (PSM).

There were no statistically significant differences in sex or mean gestational age between the two groups (**Table 1**). Group 2 had a significantly lower birth weight than Group 1 (809.57±264.10 grams vs. 922.37±263.35 grams, p <0.001). Hence, the proportion of infants with SGA was significantly higher in Group 2 (21.69%) than in Group 1 (6.09%) (p <0.001). The two study groups had no difference in the Apgar score at 1 min and 5 min after birth. Group 1 had a significantly lower rate of PDA (42.81% vs. 57.83%, p = 0.001) and ASD (5.92% vs. 10.84%, p = 0.028) than Group 2. The proportion of infants with PDA who underwent ligation was similar in both groups. No significant difference in perinatal bacterial sepsis was observed between the two groups. More babies in Group 1 were treated with surfactant replacement therapy (Survanta) than in Group 2 (p = 0.033). More infants in Group 2 received combined use of other inotropes than in Group 1 (dobutamine, dopamine, and epinephrine, p <0.001). The duration of dobutamine use was higher in Group 2 than in Group 1 (p = 0.0453) (**Fig 3A**).

After PSM, each group’s sample size is 124. SGA and ASD became insignificant between the two study groups. PDA was a borderline difference between the two study groups, but there was no difference in the proportion of infants with PDA who underwent surgical ligation. The timing of PDA ligation for Group 1 and Group 2 was 2.46 ±7.95 days of age and 1.18±3.19 days of age, respectively (p = 0.312). More infants in Group 2 using inotropes than in Group 1 remained (**Table 1**). However, the duration of combined use of these three inotropes was similar between the two study groups (**Fig 3B**).

### Mortality

**Fig 4:**
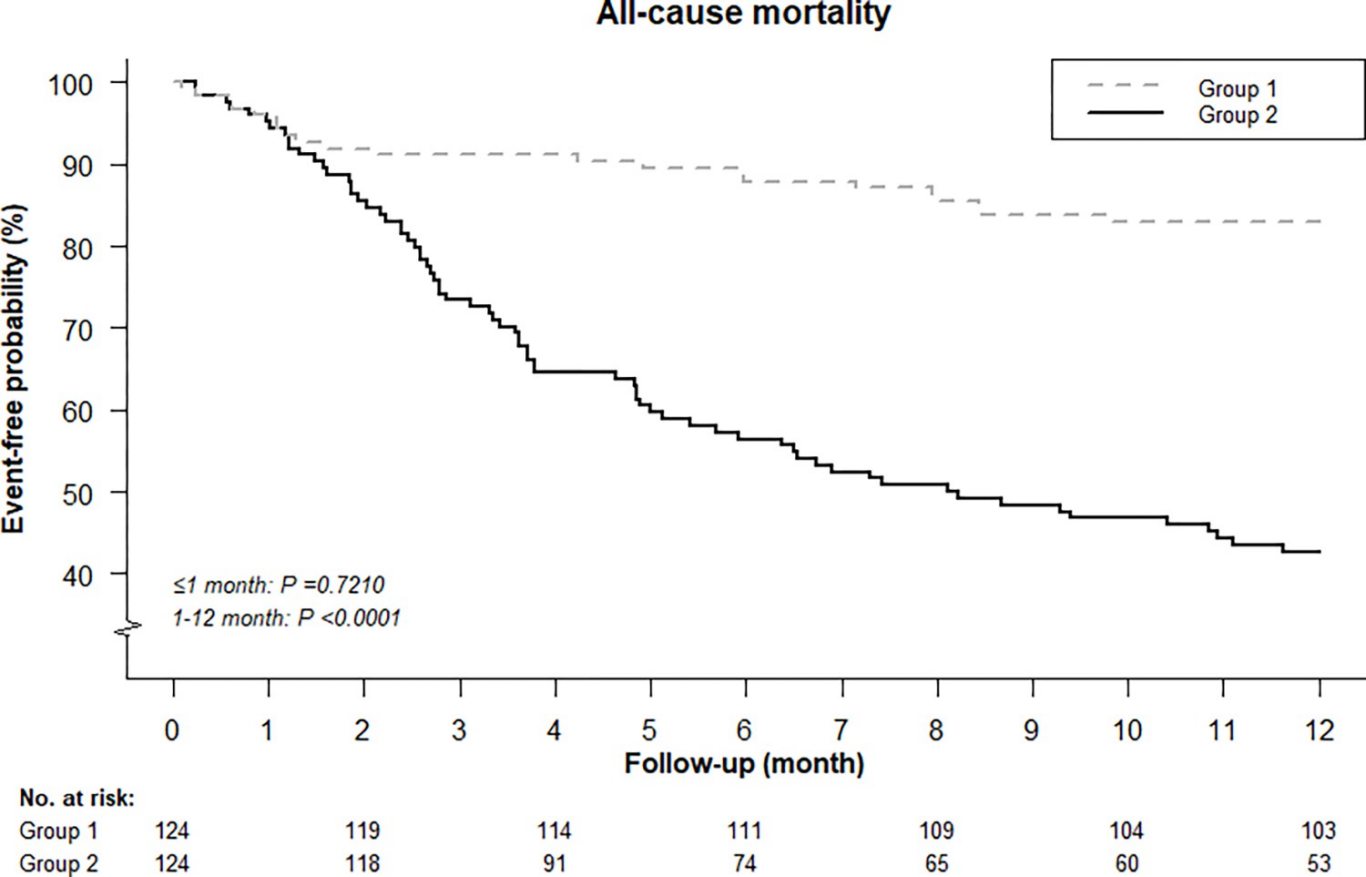
Kaplan-Meier curves of all-cause mortality among very low birth weight preterm infants treated with inhaled nitric oxide after propensity score matching (PSM).

After PSM, there were 21 (16.9%) and 71 (57.3%) deaths for Groups 1 and 2, respectively. The all-cause mortality within one year of age (**Fig 4**) was significantly higher in Group 2 than in Group 1 (p<0.001). The main difference in mortality between the two study groups occurred one month after birth (55.1% vs. 13.5%), and the adjusted HR of all-cause mortality for Group 2 from month 2 to year 1 was 4.25 (95%CI = 2.42–7.47) compared with Group 1.

### Respiratory condition

Most of the VLBWP infants received iNO starting from birth (n = 119 (96.0%), 116 (93.5%) for Group 1 and Group 2, respectively). The mortality rate at 36 weeks of postmenstrual age (PMA) was 8.9% for Group 1 and 16.1% for Group 2. Hence, the data regarding various ventilatory support was stratified by death or survival till 36 weeks PMA. For VLBWP infants who died before 36 weeks PMA, the length of hospital stay in Group 2 was significantly longer than that in Group 1. Accordingly, the days of iNO use and the days of HFOV use were significantly longer in Group 2 compared with Group 1. The use of iNO seemed to be persistent until the death. There was no difference in the incidence of moderate and severe BPD between the two groups (p = 0.128).

For VLBWP infants who survived after 36 weeks PMA, the length of hospital stay, duration of iNO use, and days requiring invasive conventional mechanical ventilation between the two study groups were similar. On the other hand, more infants required HFOV, but fewer infants needed non-invasive ventilation and supplemental oxygen in Group 2 than those in Group 1. The duration of non-invasive ventilation was significantly longer in Group 2 than in Group 1. There was no difference in the incidence of overall BPD between the two groups, but the incidence of moderate and severe BPD was significantly higher in Group 2 compared to Group 1 **(Table 2).**

**Table 2:** The respiratory condition among very low birth weight preterm infants treated with inhaled nitric oxide after propensity score matching (PSM), by death before or survival after 36 weeks PMA.

	Death before 36 weeks PMA					Survival after 36 weeks PMA				
	Group 1	(n = 11)	Group 2	(n = 20)	P	Group 1	(n = 113)	Group 2	(n = 104)	P
**Length of hospital stay**										
Mean ±SD (days)	16.91	±10.18	31.15	±23.16	0.025	75.35	±26.72	79.20	±33.77	0.355
Medium (min-max)	15.00	(3–33)	27.50	(4–83)		68.00	(27–203)	68.00	(19–201)	
**After intubation**										
** Inhaled nitric oxide (iNO) usage**										
Mean ±SD (days)	16.91	±10.18	31.15	±23.16	0.025	70.99	±25.47	75.98	±33.17	0.218
Medium (min-max)	15.00	(3–33)	27.50	(4–83)		62.00	(26–194)	66.00	(19–201)	
**High-frequency oscillatory ventilation (HFOV)**										
Yes (%)	6	(54.55%)	14	(70.00%)	0.390	54	(47.79%)	76	(73.08%)	<0.001
Mean ±SD (days)	16.17	±10.11	38.64	±22.10	0.030	63.33	±14.60	67.99	±22.56	0.156
Medium (min-max)	16.00	(3–33)	41.00	(6–83)		61.00	(26–108)	64.00	(22–140)	
**Invasive conventional mechanical ventilation**										
Mean ±SD (days)	16.91	±10.18	31.15	±23.16	0.025	69.17	±23.97	71.63	±27.43	0.482
Medium (min-max)	15.00	(3–33)	27.50	(4–83)		62.00	(17–194)	65.50	(16–160)	
**Without intubation**										
**Non-invasive ventilation**										
Yes (%)	1	(9.09%)	3	(15.00%)	1.000	108	(95.58%)	77	(74.04%)	<0.001
Mean ±SD (days)			63.33	±23.71	-	67.30	±19.85	77.02	±33.13	0.024
Medium (min-max)	26.00	(26–26)	70.00	(37–83)		61.00	(27–155)	64.00	(31–201)	
**Supplemental oxygen via nasal cannulas**										
Yes (%)	0	(0.00%)	0	(0.00%)	-	97	(85.84%)	48	(46.15%)	<0.001
Mean ±SD (days)					-	54.93	±25.15	61.21	±33.18	0.744
Medium (min-max)						58.00	(2–146)	61.50	(5–177)	
Bronchopulmonary dysplasia (BPD)	2	(18.18%)	10	(50.00%)	0.128	112	(99.12%)	102	(98.08%)	0.608
Moderate and severe BPD	2	(18.18%)	10	(50.00%)	0.128	51	(45.13%)	87	(83.65%)	<0.001

Group 1, without milrinone use; Group 2, with milrinone use; SD, standard deviation.

### Secondary outcomes

**Fig 5:**
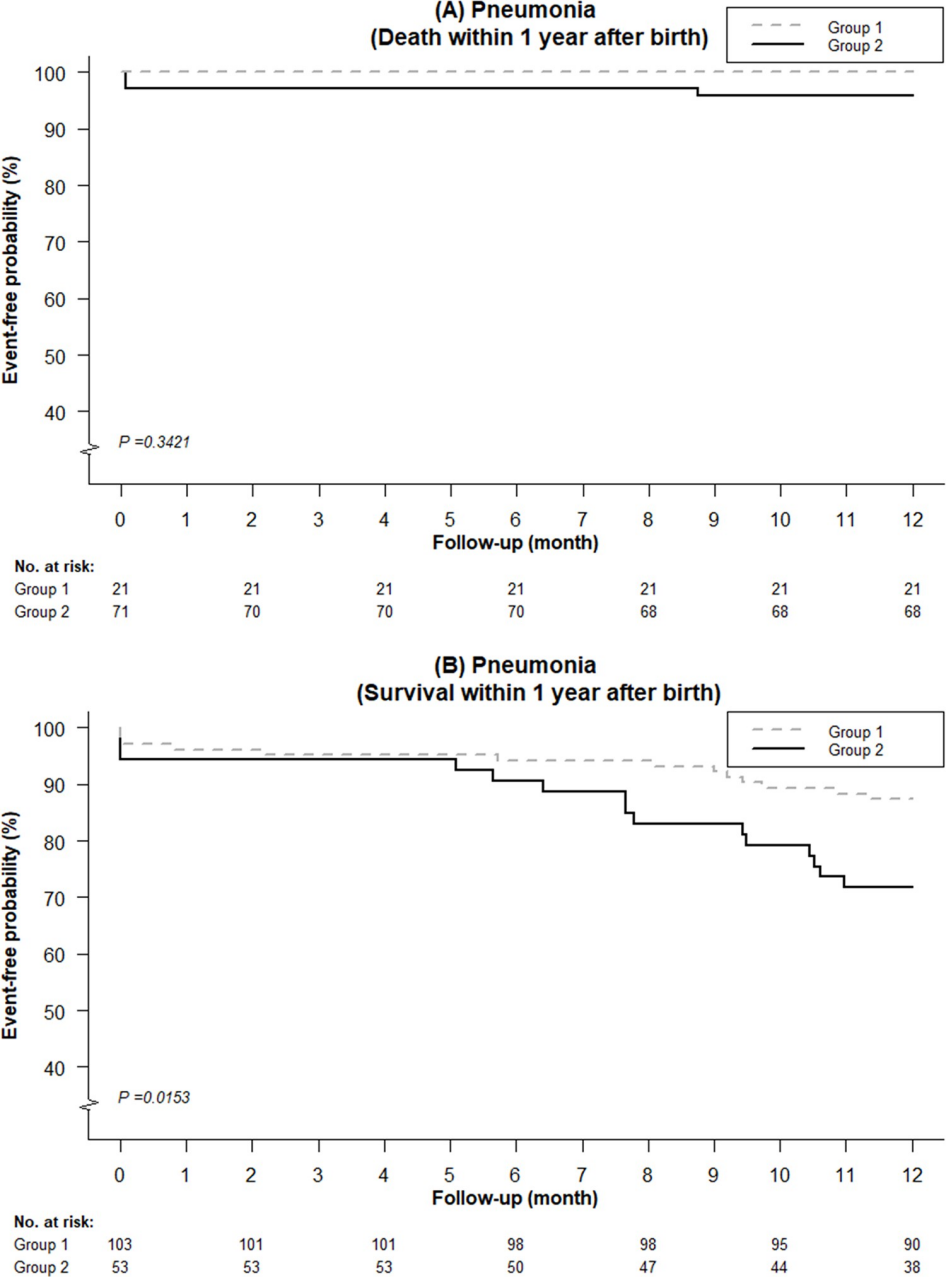
Kaplan-Meier curves of pneumonia among very low birth weight preterm infants treated with inhaled nitric oxide after propensity score matching. (A) For infants who died within one year after birth, and (B) for infants who survived till one year of age.

**Fig 6:**
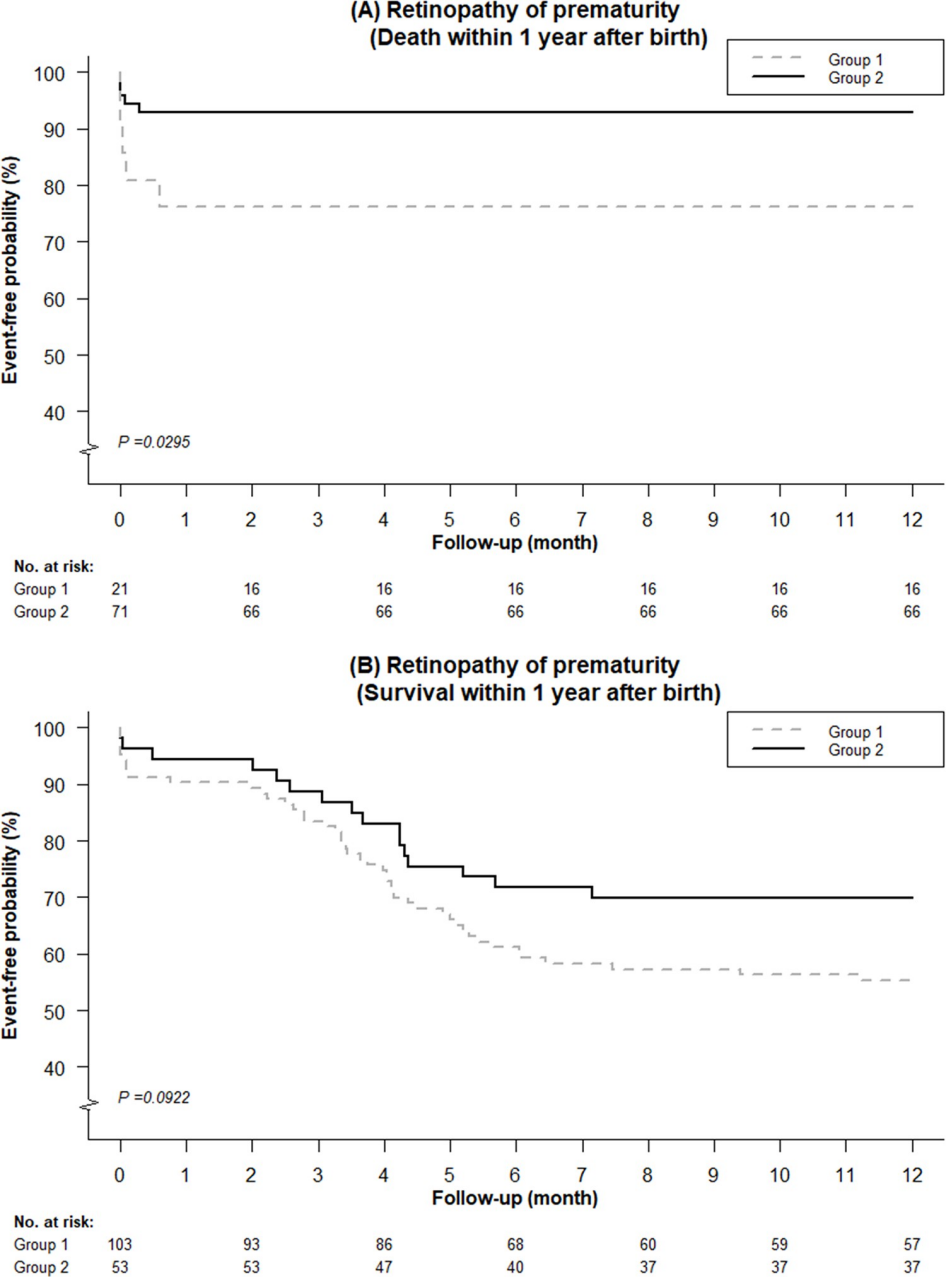
Kaplan-Meier curves of retinopathy of prematurity among very low birth weight preterm infants treated with inhaled nitric oxide after propensity score matching. (A) For infants who died within one year after birth, and (B) for infants who survived till one year of age.

There were no secondary outcomes occurring before 36 weeks PMA, except three babies in Group 2 having necrotizing enterocolitis. Hence, the data regarding preterm-associated morbidities was further stratified by death or survival till one year of age, instead of 36 weeks PMA (**Table 3**). Regarding infants who died prior to one year of age, no difference in IVH was found between the two groups. For infants in Group 2, 9.86%, 1.39%, and 4.23% had NEC, late-onset sepsis, and pneumonia, respectively, whereas no infants in Group 1 had these morbidities. There was no statistical significance for the pneumonia rate between the two groups (p = 0.3421) **(Fig 5A)**. The incidence of ROP remained significantly lower in Group 2 compared to Group 1 (23.81% vs. 7.04%, p = 0.032 after considering the competing risk of death) **(Fig 6A)**. Among infants who were alive till one year of age, no differences in the incidence of IVH, NEC, epilepsy, and late-onset sepsis were observed between the two groups. The incidence of pneumonia was significantly higher in Group 2 compared to Group 1 (p = 0.0153) **(Fig 5B)**. On the other hand, the incidence of ROP in Group 2 was lower than in Group 1, but it was not statistically significant (p = 0.0922) **(Fig 6B)**.

**Table 3:** The secondary outcomes among very low birth weight preterm infants treated with inhaled nitric oxide after propensity score matching (PSM), by death or survival within one year after birth.

	Death within 1 year after birth					Survival at 1 year after birth			
	Group 1 (n = 21)		Group 2 (n = 71)			Group 1 (n = 103)		Group 2 (n = 53)	
	n	%	n	%		n	%	n	%
**(A) Early outcomes**									
Intraventricular hemorrhage	1	4.76%	2	2.82%		6	5.83%	3	5.66%
OR (95% CI), P	0.58 (0.05–6.73), 0.663					0.97 (0.23–4.04), 0.967			
Adjusted OR (95% CI), P	0.52 (0.04–6.30), 0.609					0.75 (0.17–3.24), 0.700			
Necrotizing enterocolitis	0	0.00%	7	9.86%		4	3.88%	1	1.89%
OR (95% CI), P	-					0.48 (0.05–4.37), 0.512			
Adjusted OR (95% CI), P	-					0.39 (0.04–3.76), 0.413			
**(B) First-year outcomes after birth**									
Epilepsy	0	0.00%	0	0.00%		5	4.85%	4	7.55%
HR (95% CI), P	-					1.57 (0.42–5.85), 0.501			
Adjusted HR (95% CI), P	-					1.14 (0.30–4.28), 0.850			
Late-onset sepsis	0	0.00%	1	1.39%		6	5.83%	2	3.77%
HR (95% CI), P	-					0.65 (0.13–3.22), 0.597			
Adjusted HR (95% CI), P	-					0.64 (0.13–3.23), 0.586			
Pneumonia	0	0.00%	3	4.23%		13	12.62%	15	28.30%
HR (95% CI), P	-					2.44 (1.16–5.12), 0.019			
Adjusted HR (95% CI), P	-					2.44 (1.14–5.23), 0.022			
Retinopathy of prematurity (ROP)	5	23.81%	5	7.04%		46	44.66%	16	30.19%
HR (95% CI), P	0.28 (0.08–0.94), 0.030					0.62 (0.35–1.09), 0.095			
Adjusted HR (95% CI), P	*0.31 (0.11–0.90), 0.032					0.65 (0.37–1.16), 0.148			

Group 1, without milrinone use; Group 2, with milrinone use; HR, the hazard ratio for outcomes within one year (epilepsy, late-onset sepsis, pneumonia, and retinopathy of prematurity); OR, the Odds ratio for early outcomes (intraventricular hemorrhage and necrotizing enterocolitis).

Adjusted OR/HRs were adjusted for using inotropes (dobutamine, dopamine, epinephrine).

*After competing risk analysis.

## Discussion

This retrospective study revealed that iNO treatment in VLBWP infants increased from 3.3% to 8.4% in Taiwan during 2004–2019, higher than that reported in the United States [26]. Most iNO treatments start from birth and last for more than one month. Three of four infants treated with iNO need inotropic use concomitantly, and concurrent use of iNO and intravenous milrinone infusion was increased to one-third of cases in 2019. The possible explanation for the escalated use of iNO with milrinone could be due to an inadequate response to iNO or the occurrence of pulmonary hypertension with right ventricular dysfunction [27]. In this study, PDA and SGA were two previously known risk factors for the concurrent use of iNO and milrinone. To reduce the difference between the two groups, PSM was performed without adjusting the use of inotropes, and therefore the frequency of inotropes use was higher in Group 2. The proportion of hemodynamic instability may be higher in VLBWP infants treated with both iNO and milrinone. In the analysis of secondary outcomes (**Table 3**), the results before and after adjusting inotropes use were similar. This suggests that inotropes use (dobutamine, dopamine, and epinephrine) has little effect on secondary outcomes.

In this study, death prior to 36 weeks PMA occurred in eleven infants in Group 1 (8.9%) and twenty infants in Group 2 (16.1%), much lower than in other countries. For instance, a study in England reported that 18% of total extremely preterm infants died before 36 weeks PMA during 2014–2018 [28]. The retrospective study in the United States showed that 11% of extremely preterm infants died before hospital discharge, and the mortality is highest in the first 3 months after birth [29, 30]. In Taiwan, there is no definite protocol or regulation of end-of-life practices for VLBWP infants with hypoxemic respiratory failure. Most neonatologists follow international guidelines [30] and make decisions with the parents together on withdrawing or withholding life-sustaining treatment. Furthermore, clinical decisions regarding when to elective discontinuation of ongoing life support may vary between different neonatal intensive care units (NICUs). The delay in withdrawing life support for VLBWP infants with hypoxemic respiratory failure is likely in Taiwan because of the attitude towards life and all expenses covered by the national health insurance program. Hence, the overall death among VLBWP infants in Taiwan occurred later than in other countries.

Notably, the significant difference in mortality between the two groups from month 2 to year 1 was observed in our national cohort. Because of very few pharmacokinetic and pharmacodynamic studies on milrinone use in VLBWP infants, we cannot conclude that late death in Group 2 was related to milrinone use. In a previous retrospective cohort study, milrinone resulted in a fall in blood pressure after 6 hours, and inotropes were required to maintain adequate blood pressure [27]. Similar hypotensive events were observed in term infants with pulmonary hypertension treated with iNO and milrinone [31]. Even though it has been observed that milrinone may induce systemic vascular hypotension, no severe complications or milrinone-related mortality were reported. We believe that the late death in Group 2 is not due to milrinone use, but the severe hypoxemic respiratory failure per se contributes to higher mortality. The difference in severe hypoxemia between the two groups may already exist right after birth.

In addition, the high mortality rate in the Group 2 may be because more VLBWP infants in the Group 2 had hemodynamic instability, considering the significant increase in inotropic use, which is likely associated with the occurrence of pulmonary hypertension [32], immature lung development, or cardiac dysfunction. Nevertheless, echocardiography was unavailable in every NICU, and the severity of pulmonary hypertension in VLBWP infants born at different gestational ages was challenging to determine. In short, the possibility to explain the late death in group 2 includes: 1) severe hypoxemia and refractory hypoxemic respiratory failure, and intravenous milrinone did not reduce severe hypoxemia; 2) pulmonary hypertension developed, and maximal use of inotropes did not stop the progression of right heart failure. The add-on milrinone use provided maximal cardiopulmonary support, probably resulting in delayed mortality but eventually did not reverse the refractory hypoxemic respiratory failure. The future prospective study may provide a more accurate outcome evaluation following the combined treatment of iNO and milrinone.

iNO was initially used to treat pulmonary hypertension in term and late preterm infants (born at ≥34 weeks of gestation). A randomized control study showed that iNO therapy significantly increased PaO_2_ and improved the oxygenation index [2]. For preterm infants with hypoxemic respiratory failure associated with pulmonary hypertension, several case series showed that iNO therapy reduced mortality and BPD [33, 34]. Selective use of iNO could be considered in this population based on recent guidelines from the AHA/ATS [35] and the Pediatric Pulmonary Hypertension Network [36]. In this study, we could not determine whether pulmonary hypertension occurred in either Group 1 or Group 2 because not all VLBWP infants had echocardiography examinations before iNO initiation.

For preterm infants with hypoxemic respiratory failure without pulmonary hypertension, there is still debate regarding using iNO [37]. A systematic review reported that iNO therapy in preterm infants did not affect mortality rates or the development of chronic lung disease [38]; therefore, routine use of iNO was not recommended for preterm infants of <34 gestation weeks who required respiratory support [39]. Although there is no evidence of a clinical benefit of iNO treatment in preterm infants with respiratory failure [12, 40], the off-label use of iNO is common, especially for VLBWP infants [41, 42]. Milrinone is an inotrope with the property of pulmonary vasodilation [43]. Combining iNO and intravenous milrinone is supposed to synergically reduce pulmonary vascular resistance to improve oxygenation in refractory hypoxemic respiratory failure. When analyzing the long-term respiratory outcomes for VLBWP infants who survived till 36 weeks PMA, the add-on treatment with intravenous milrinone did not shorten the duration of iNO use nor the days of invasive mechanical ventilation. However, following the extubation, the percentage of supplemental oxygen use via nasal cannulas was significantly reduced in Group 2. It could be possible that the concurrent use of iNO and milrinone may improve oxygenation and reduce the oxygen demand in Group 2.

In Taiwan, pre-emptive treatment with intratracheal administration of surfactant in VLBWP infants was uncommon during the study period (2004–2019), and surfactant therapy is usually applied to neonates with respiratory distress syndrome presenting increased work of breathing and radiographic findings of ground-glass opacity in lung fields. A population-based study reported that the overall frequency of surfactant therapy in infants with very low birth weight is around 40–50% [44]. In this study, most VLBWP infants received iNO starting from birth. During iNO use, it is not common to disconnect iNO and then administer intratracheal surfactant. It is reasonable to observe lower rate of surfactant use in our study compared to the previous report in Taiwan. Clinically, it could be possible that clinicians did not use surfactant immediately after birth based on the clinical conditions and radiographic findings. However, when the hypoxemic respiratory failure got worse, iNO was chosen, possibly because the clinical manifestations did not match the typical features of respiratory distress syndrome. Once iNO was used, it was not feasible to administer surfactant after that. The lower rate of surfactant use may contribute to prolonged mechanical ventilation and a higher incidence of BPD for both groups.

In this study, the incidence rates of overall BPD in Groups 1 and 2 were 91.94% and 90.32%, respectively. For infants who were alive after 36 weeks PMA, Group 2 has a significantly higher incidence of moderate and severe BPD (83.65%) than Group 1 (45.13%). The results differed from the previous study in Taiwan (the rate of severe BPD among extremely preterm infants was 56%) [45]. One possible reason is that we only studied VLBWP infants receiving iNO, and it is a small population in NICU. Another possible explanation is that the clinical criterion for diagnosis of BPD changes over time [46]. The higher rate of moderate and severe BPD in Group 2 than in Group 1 suggests that the severity of hypoxemic respiratory failure differs between the two groups. The higher rate of pneumonia in Group 2, which occurred after 36 weeks PMA, may also be associated with more incidence of moderate and severe BPD in Group 2. We speculate that the disruption of pulmonary development, especially for extremely preterm infants, plays a crucial role in BPD. Medications that reduce pulmonary vascular resistance, such as iNO therapy or intravenous milrinone, have little help in BPD prevention. For VLBWP infants with respiratory distress and progressively increased oxygen requirement, less invasive surfactant administration is suggested to apply early instead of iNO therapy [47].

Compared to the previous studies showing that the incidence of IVH in extremely preterm infants is around 15–20% [45, 48], an extraordinarily low rate of IVH in VLBWP infants was observed in this study, which might imply that iNO therapy has a neuroprotective effect in VLBWP infants. Clinical trials have described a similar effect [4, 49]. On the other hand, a large-scale meta-analysis did not support the protective effect of iNO on intracranial hemorrhage [50]. The concentration of iNO, the timing of iNO initiation, and the duration of iNO therapy may make differences, and further detailed investigations are warranted. Cerebral blood flow disturbance is crucial in developing IVH [51]. In VLBWP infants, dynamic autoregulation of cerebral blood flow could not yet be fully developed [52]. Tissue hypoxia is a risk factor for developing IVH, possibly related to the fluctuation of cerebral blood flow [53, 54]. A previous study showed that the burden of cerebral hypoxia was significantly higher in extremely preterm infants with IVH than those without [54]. In contrast, the recent randomized, phase 3 trial showed that intervention guided by cerebral oximetry monitoring to normalize cerebral oxygenation did not reduce severe brain injury [55]. Whether iNO therapy improved cerebral tissue hypoxia or reduced fluctuation of cerebral blood flow was not documented in this retrospective study. Because the sample size of this study is small, the extraordinarily low rate of IVH is probably a chance finding.

In this study, the incidence rate of perinatal bacterial sepsis was around 25–30% (**Table 1**), whereas late-onset sepsis rate was 3.77–5.83% (**Table 3**). The recent large-scale studies obtained from a nationally representative cohort in the United States showed early-onset and late-onset sepsis incidence rates were 1.4% and 8.9%, respectively [56, 57]. The perinatal condition of the VLBWP infants receiving iNO therapy in our study could differ greatly from the US cohort mentioned above. The possible reasons for the very low rate of late-onset sepsis in our study would be antibiotic treatment already used for the treatment of early-onset perinatal sepsis, fluconazole prophylaxis, the introduction of probiotics, and improved bundle care of central venous catheter in the NICUs in Taiwan. Another reason for the low rate of late-onset sepsis in this study is that our study groups were VLBWP singletons with hypoxemic respiratory failure treated with iNO, which cannot represent the whole VLBWP infants.

ROP is a common complication in VLBWP infants. To the best of our knowledge, the effect of pulmonary vasodilators on retinal development has not yet been determined. The role of NO in the pathogenesis of ROP is multifactorial; for example, NO may contribute to or reduce retinal neovascularization, depending on the different isoforms of NO synthase and the amount of NO produced [58, 59]. The meta-analysis conducted by Yang et al. reported no elevated risk of ROP in preterm infants treated with iNO [50]. In a retrospective case-control study, there was no effect on ROP progression in preterm infants born before 30 weeks of gestational age with sildenafil treatment for a median duration of 52 days [60]. In this study, there was no deterioration of ROP following milrinone treatment. In contrast, for infants who died prior to one year of age, the incidence of ROP in Group 2 was significantly lower compared to Group 1. For infants who were alive within one year of age, there is a trend toward lower risk of ROP in Group 2 but did not reach statistical significance. Future animal experiments and large-scale prospective studies are needed to determine the effect of the combined use of iNO and milrinone on the development of ROP.

The strengths of this study are its representative and relatively large sample size. Because the annual rate of VLBWP singleton infants is minimal (about 4 per 1,000 newborns based on our data) and the proportion of ever-use iNO after birth (4.6% based on our data), it took 16 years for Taiwan with an annual 200,000 newborns to observe 591 VLBW infants in Group 1 and 166 VLBW infants in Group 2. The two primary outcomes (use of high-frequency oscillatory ventilation and the duration involved; use of non-invasive ventilation and the duration involved) are important clinically. Our data has enough statistical power (99%) to detect the difference in high-frequency oscillatory ventilation between the two study groups (48.4% vs. 72.6%). For those who needed high-frequency oscillatory ventilation, we need 1500 VLBW infants in Group 1 and 2000 VLBW infants in Group 2 with a statistical power of 80% to detect their duration difference. Moreover, our data has enough statistical power to detect the difference in using non-invasive ventilation (99%) and the duration involved (92%) between the two study groups.

On the other hand, this study has some limitations. (1) Our findings may be subject to indication bias because clinicians tend to administer intravenous milrinone for VLBWP infants with poor response to iNO alone or add milrinone to hemodynamic instability. Hence, it is reasonable to see a higher proportion of PDA and SGA in Group 2 than in Group 1 before PSM. After PSM without adjusting the inotropes, the frequency of inotropic use was higher in Group 2. The clinical condition may be more critical in Group 2, resulting in less favorable outcomes. (2) Previous studies reported that approximately 8–18% of VLBWP infants develop pulmonary hypertension [61, 62]. No information was available regarding the oxygenation index, hemodynamic data, or echocardiographic findings. Therefore, we could not evaluate the occurrence and severity of pulmonary hypertension before and after iNO and milrinone infusion for both groups. (3) We could not evaluate whether concurrent use of iNO and intravenous milrinone directly contributes to hemodynamic instability or other adverse effects because these clinical parameters were unavailable in NHIRD. (4) Our results may not generalize to other populations, including VLBWP infants with diverse ethnicities or treated with different mechanical ventilation strategies.

In conclusion, most VLBWP infants treated with iNO had inotropic support concomitantly. Combination treatment of iNO and intravenous milrinone did not shorten the duration of ventilation support. Compared to the VLBWP infants who received iNO only, infants who need concurrent use of iNO and milrinone have a higher proportion of developing moderate and severe BPD and a lower survival rate during the first-year follow-up, possibly because the severity of hypoxemic respiratory failure is worse. Medications that facilitate pulmonary vasodilation based on physiologic rationale did not improve one-year outcomes of hypoxemic respiratory failure in VLBWP infants. However, the 30-day survival rate of concurrent use of milrinone was similar to iNO treatment alone, suggesting short-term benefit exists. The optimal treatment for VLBWP infants with refractory hypoxemic respiratory failure has not been established. This study provides a foundation for a future prospective study to evaluate the benefits of concurrent iNO and milrinone use in VLBWP infants.

## Supporting information

S1 Table Codes for comorbidities and outcomes. (DOCX)

S2 Table Codes for treatments or medications. (DOCX)
