# Supplementary material for: First-year outcomes of very low birth weight preterm singleton infants with hypoxemic respiratory failure treated with milrinone and inhaled nitric oxide (iNO) compared to iNO alone: A nationwide retrospective study
Source: PLoS One. 2024 May 9;19(5):e0297137. doi: 10.1371/journal.pone.0297137 (PMC11081351; doi:10.1371/journal.pone.0297137)
Supplement: S1 Table — (DOCX) [file pone.0297137.s001.docx]

|  | **ICD9-CM codes** | **ICD-10-CM codes** | **Extra criteria** |
| --- | --- | --- | --- |
| Patent ductus arteriosus | 747.0 | Q25.0 | Hospitalization once, or outpatient visit twice or more |
| Atrial septal defect | 745.5 | Q21.1 | Hospitalization once, or outpatient visit twice or more |
| Perinatal bacterial sepsis | 771.8 | P36, P39, R78.81 | Hospitalization once, or outpatient visit twice or more |
| Intraventricular hemorrhage | 772.1 | P52.3 | Hospitalization once, or outpatient visit twice or more |
| Necrotizing enterocolitis | 777.50 | P77.9 | Hospitalization once, or outpatient visit twice or more |
| Epilepsy | 345 | G40.1-G40.5, G40.8, G40.9 | Hospitalization once, or outpatient visit twice or more |
| Late-onset sepsis | 0389 | R65.1, R65.2, A41.9 | Hospitalization once, or outpatient visit twice or more |
| Retinopathy of prematurity | 362.2 | H35.109, H35.119, H35.129, H35.139, H35.149, H35.159. H35.169, H35.179, H35.23 | Hospitalization once, or outpatient visit twice or more; and received ventilator use at least 28 days |
| Pneumonia | 481-483 | A48.1, J13, J14, J15.0, J15.1, J15.20, J15.211, J15.212, J15.29, J15.3, J15.4, J15.5, J15.6, J15.7, J15.8, J15.8, H15.9, J16.0, J16.8, J18 | Hospitalization once, or outpatient visit twice or more |
