# Supplementary material for: First-year outcomes of very low birth weight preterm singleton infants with hypoxemic respiratory failure treated with milrinone and inhaled nitric oxide (iNO) compared to iNO alone: A nationwide retrospective study
Source: PLoS One. 2024 May 9;19(5):e0297137. doi: 10.1371/journal.pone.0297137 (PMC11081351; doi:10.1371/journal.pone.0297137)
Supplement: S2 Table — (DOCX) [file pone.0297137.s002.docx]

| **Treatments or medications** | **Taiwan NHI codes** |
| --- | --- |
| Patent ductus arteriosus ligation | 69031B, 69031A |
| Survanta | B019595426 |
| Dobutamine | A032381238, A038284238, A038707238, A042141238, A046298238, AC38284238, AC42141238, AC46298238, AC58566238, B015836265, B016731221, B016819220, B016819238, B021004238, B021217238, B021570221, B021826238, B021957238, B021965228, B021965238, B023800221, B023870238, BC23800221, A049901263, AC49901263, A049901265, AC49901265, A049901277, AC49901277, AC58770265 |
| Dopamine | B017008221, B014442219, A021978221, A032704221, A032792229, A042276221, A044090221, A049314221  AC21978221, AC32704221, AC44090221, B006378229, B007508221, B015177221, B015749221, B016157221, B018632221, B021687221, B025227221, BC25227221, B017024229, B020605229, B015749229, A032705221, B005671221, B018631221, A030765277, B015397277, B023341277, B023341265, BC23341265, A049695265, A049695277, A049624265, AC49695265, B015398265, B023340265, BC23340265, AC49695277, B022262263, A055539265, A055539277, AC55539265, B023361265  BC23361265, AC55539277, A046457263, AC46457263, B022261263 |
| Epinephrine | N004065355, A000480209, A015514209, A033761209, AC00480209, AC15514209, AC33761209, AC57890209, B018183209, B019413229, N004066209 |
